# *ERCC1* and *CYP1B1* polymorphisms as predictors of response to neoadjuvant chemotherapy in estrogen positive breast tumors

**DOI:** 10.1186/s40064-015-1053-0

**Published:** 2015-07-07

**Authors:** Aurélie Dumont, Diane Pannier, Agnès Ducoulombier, Emmanuelle Tresch, Jinying Chen, Andrew Kramar, Françoise Révillion, Jean-Philippe Peyrat, Jacques Bonneterre

**Affiliations:** Laboratoire d’Oncologie Moléculaire Humaine, Centre Oscar Lambret, 3 rue Frédéric Combemale, BP 307, 59020 Lille Cedex, France; Département de Sénologie, Centre Oscar Lambret, 3 rue Frédéric Combemale, BP 307, 59020 Lille Cedex, France; Unité de Méthodologie et Biostatistique, Centre Oscar Lambret, 3 rue Frédéric Combemale, BP 307, 59020 Lille Cedex, France

**Keywords:** Breast cancer, Neoadjuvant chemotherapy, *ERCC1*, *CYP1B1*, Single nucleotide polymorphism, Predictive factor

## Abstract

**Purpose:**

Neoadjuvant chemotherapy (NCT) using anthracyclines and taxanes is a standard treatment for locally advanced breast cancer. Efficacy of NCT is however variable among patients and predictive markers are expected to guide the selection of patients who will benefit from NCT. A promising approach stand with polymorphisms located in genes encoding drug transporters, drug metabolizing enzymes and target genes which can affect drug efficacy. Our study investigated the potential of 37 polymorphisms to predict response to NCT in breast cancer.

**Methods:**

118 women with breast adenocarcinoma were treated with FEC100 and taxotere. Genotyping was performed on germline DNA using the BioMark platform (Fluidigm). Pathological complete response (pCR) according to Sataloff criteria was correlated to clinical characteristics and genotypes using univariate and multivariate analyses.

**Results:**

25 patients (21.2%) reached complete pathologic response. pCR rate is increased in SBRIII (p = 0.009), ER negative (p = 0.005) and triple negative (p = 0.006) tumors. pCR rate is significantly increased for patients carrying at least one variant allele for *BRCA1*, *ERCC1* or *SLCO1B3,* and for patients homozygous for *CYP1B1*. The combination of *ERCC1* and *CYP1B1* polymorphisms is a potential predictor of NCT response in breast cancer (pCR rate reached 50 vs 21.2% for unselected patients), and particularly in ER + breast cancer subtype where pCR rate reached 41.2 vs 13.5% for unselected patients.

**Conclusions:**

This study is the first to report *ERCC1*, *BRCA1* and *SLCO1B3* as markers of response to NCT in breast cancer. *ERCC1*/*CYP1B1* combination might be of particular interest to predict response to NCT in breast cancer and particularly to help NCT indication for ER+ breast tumors.

**Electronic supplementary material:**

The online version of this article (doi:10.1186/s40064-015-1053-0) contains supplementary material, which is available to authorized users.

## Background

Neoadjuvant chemotherapy (NCT) improves clinical outcome as patients whose tumors respond to NCT have superior disease-free and overall survival than patients whose tumors do not (Rastogi et al. 2008). Thus, predicting the chance of response before starting NCT is crucial to identify patients who will benefit from NCT and help to select for the appropriate drugs. Up to now, several markers such as tumor size, histological type, hormone receptor status, and HER2 expression are used to predict efficiency of NCT. However, despite the consideration of these tumor characteristics, heterogeneity in therapy efficacy still subsists and accurate predictive markers for NCT are lacking.

Genetic variations such as single nucleotide polymorphisms (SNPs) are present across individuals and might affect pharmacokinetics and pharmacodynamics of drugs. SNPs in genes encoding drug transporters, drug metabolizing enzymes and target genes can directly affect the drug uptake, its activation and its excretion, accessibility of the target and also related pathways that can potentiate its action. Consequently, SNPs might lead to therapeutic failures and/or adverse drug reactions. Currently, UDP glucuronosyltransferase 1A1polymorphism UGT1A1*28 is used to predict toxicity of irinotecan in colorectal cancer treatment. Genotyping allows individuals homozygous for the variant allele of *UGT1A1* to be treated with reduced doses of irinotecan and to avoid severe toxicity.

The aim of our study was to identify SNPs as predictive markers for breast cancer NCT. In our institution, breast cancer women treated with NCT receive 5-FU, cyclophosphamide, anthracyclines and/or taxanes. Thus, we selected SNPs previously reported in the literature to be associated with response to these chemotherapeutic agents. Thirty-seven SNPs, located in genes encoding influx and efflux transporters such as *SLCO1B3*, *MDR1* and *ABCC4*, in genes belonging to drug metabolism (*CYP1B1*, *CYP2B6*, *CYP3A4*, *MTHFR* and *GSTP1*…) and also in genes of DNA repair pathway such as *XRCC1*, *ERCC1*, *BRCA1* and *p53* (Additional file 1: Table S1) were investigated in breast cancer patients receiving NCT.

## Patients and methods

### Patients

191 women with histological proven breast adenocarcinoma were enrolled in the study between November 2007 and January 2012 (ClinicalTrials.gov identifier: NCT00959556). Patients were treated with anthracyclines and/or taxanes based NCT in our institution (Centre Oscar Lambret, Lille, France). Among them, 118 received FEC100-Taxotere, 46 patients with HER2+ tumors were treated with FEC100-Taxotere-Herceptin, 16 patients were treated with FEC100 and 11 patients received diverse chemotherapy regimens.

Patient characteristics are summarized in Table 1 for the two main groups. Median age at diagnosis was 46.5 years for patients treated with FEC100-taxotere and 45.5 years for patients treated with FEC100-taxotere-herceptin. The median size of the tumors was 30 mm for patients treated with FEC100-taxotere and 30.5 mm for patients treated with FEC100-taxotere-herceptin. The histoprognostic grading was established according to Scarff Bloom and Richardson modified by Elston and Ellis (1998). FEC100-Taxotere was mostly administrated during 6 cycles (84.7%) and the majority of patients (82.2%) received 3 cycles of FEC100 followed by 3 cycles of Taxotere. The median number of treatment cycles of Herceptin per patient was 18 (3–18).

**Table 1:** Characteristics of patients treated with FEC100-taxotere and FEC100-taxotere-herceptin

	FEC100-taxotere		FEC100-taxotere-herceptin	
	N = 118	%^a^	N = 46	%^a^
*Tumor type*				
Ductal carcinoma	106	89.9	44	95.6
Lobular carcinoma	7	5.9	1	2.2
Other	5	4.2	1	2.2
*Tumor size*				
T0-T1	5	4.3	1	2.3
T2	60	51.7	21	48.8
T3	24	20.7	11	25.6
T4	27	23.3	10	23.3
NA	2	1.7	3	6.5
*Histoprognostic grade*				
SBRI	7	6.7	1	2.5
SBRII	54	51.9	19	47.5
SBRIII	43	41.3	20	50
NA	14	11.9	6	13.1
*Nodal status*				
Positive	63	54.3	30	65.2
Negative	53	45.7	16	34.8
NA	2	1.7	0	0
*Hormone receptor status*				
Positive^b^	77	66.3	23	50.0
Negative^c^	39	33.6	23	50.0
NA	2	1.7	0	0.0
*HER-2 status*				
Positive^d^	5	4.7	46	100.0
Negative	102	95.3	0	0.0
NA	11	9.3	0	0.0
*Clinical response*				
CR	11	12.2	7	21.9
PR	62	68.9	23	71.9
SD	15	16.7	2	6.3
PD	2	2.2	0	0.0
NA	28	23.7	14	30.4
*pCR*				
Yes	25	21.2	18	40.0
No	93	78.8	27	60.0
NA	0	0.0	1	2.2

*CR* complete response, *PR* partial response, *SD* stable disease, *PD* progressive disease, *NA* not applicable, *pCR* pathological complete response.

^a^Percentages of evaluable patients (except for NA: percentage of all patients).

^b^Estrogen receptor or progesterone receptor positive.

^c^Estrogen receptor and progesterone receptor negative.

^d^IHC3+ or IHC2+/FISH+ or CISH+.

Our study only focused on patients treated with FEC100-Taxotere (n = 118), in order to identify potential predictive polymorphisms in a homogeneous group according to treatment.

### Clinical response assessment

Clinical evaluation of response to therapy was assessed by measuring tumor size (physical examination and ultrasonography) before NCT, and after 3 and 6 cycles. Change in tumor size was determined by comparing the tumor size before and after NCT according to RECIST guidelines, version 1.1 (Eisenhauer et al. 2009).

### Pathological response assessment

Tumor response was assessed by a pathologist and graded according Sataloff et al. (1995). Pathological tumor response was used as the gold standard to evaluate treatment response. On the basis of patients’ pathological response in primary breast site and/or in the lymph nodes, patients were designated having a pCR when surgical samples showed total or near-total effect and absence of nodal involvement (TA and NA or NB).

### Hormone receptors and HER2 assessment

Immunohistochemistry (IHC) was used for evaluation of oestrogen receptors (ER) (1D5 Dako clone, then SP1 Ventana clone) and progesterone receptors (PR) (1A6 Dako clone, then PR88 Biogenex clone); to be positive, more than 10% of the tumor nucleus cells had to be stained. Evaluation of HER2 was performed with IHC (CB11 Biogenex clone, then HER 485 Dako clone); only tumors classified 3+ were considered positive. All tumors HER2 2+ with IHC were tested by FISH. Triple negative tumors were defined as ER and PR negative and HER2 negative by immune histochemistry (IHC) or by FISH in case of HER2 2+.

### DNA extraction

Genomic DNA was extracted from EDTA-treated blood samples (300 µl) using the MagNA Pure Compact instrument (Roche Diagnostics, Meylan, France) and the MagNA Pure Compact Nucleic Acid Isolation Kit according to the manufacturer’s instructions.

### Genotyping

SNP genotyping was assessed using allelic discrimination with SNPType assays ordered from Fluidigm. When SNPType assays were not available, TaqMan assays (Life Technologies) were used instead. Because DNA concentration available was below supplier recommendation, a Specific Target Amplification was performed to enrich targeted SNP sequences according to the supplier’s instructions.

#### Instrumentation and nanofluidic chips

48.48 Dynamic Array used in the present study are nanofluidic chips able to analyze 48 samples with 48 SNP assays on the BioMark platform (Fluidigm). The BioMark system is used to thermal cycle these nanofluidic chips and image the data in real time.

#### SNP genotyping using SNPType assays

3 µL of SNPType ASP1/ASP2 (allele specific primer 1 and 2) and 8 µL of SNPtype LSP were premixed and dilute with 29 µL of DNA suspension buffer to prepare SNPType assay mix. Each assay (5 µL) comprised 1 µL SNPType assay mix, 2.5µL assay loading reagent 2X (fluidigm) and 1.5 µL DNA-free water. Each sample (6 µL) comprised 3 µL Biotium fast probe master mix 2X (Biotium), 0.3 µL sample loading reagent 20X (fluidigm), 0.1 µL SNPType reagent 60X (Fluidigm), 0.036 µL ROX 50X (Invitrogen), 0.064 µL DNA-free water and 2.5µL amplified genomic DNA. Each of the assays (4 µL) and samples (5 µL) was pipetted into separate inlets in the chip. Amplification was carried out under the following conditions: 95°C for 5 min, 38 cycles of 95°C for 15 s, 60°C for 45 s, 72°C for 15 s followed by 30 s at 20°C for fluorescence measurement.

#### SNP genotyping using TaqMan assays

Each assay (5 µL) comprised 2.5 µL assay loading reagent 2X (fluidigm), 0.25 µL ROX 50X (Invitrogen), 1.25 µL SNP Genotyping assay mix 40X (Applied Biosystems) and 1 µL DNA-free water. Each sample (6 µL) comprised 3 µL TaqMan universal PCR master mix 2X (Applied Biosystems), 0.3 µL sample loading reagent 20X (fluidigm), 0.3 U AmpliTaq gold polymerase (Applied Biosystems), 0.12 µL DNA-free water and 2.52 µL amplified genomic DNA. Each of the assays (4 µL) and samples (5 µL) was pipetted into separate inlets in the chip. Amplification was carried out under the following conditions: 50°C for 2 min, 98°C for 10 min, 40 cycles of 95°C for 15 s, 60°C for 60 s. Fluorescence was measured at each slice.

#### Software

The data were analyzed using the BioMark SNP Genotyping Analysis software version 3.1.2 to obtain genotype. Briefly, the software calculates the FAM, VIC or HEX fluorescence intensities relative to ROX fluorescence background, and then automatically classifies the samples into three possible genotypes.

### Statistical analysis

Clinical and histopathological characteristics were presented as frequencies and percentages for categorical variables and as medians and range for continuous variables. Associations between pCR and clinicopathological characteristics were assessed using Khi-2 test for qualitative variables or Fisher exact test in the case of small counts. Associations between pCR and continuous variables were performed using Wilcoxon Mann–Whitney test.

After ensuring that Hardy–Weinberg equilibrium was respected, the search of SNP correlated to pCR was performed by univariate logistic regression (R SNPassoc package). For each SNP, analyses were done considering genotypes separately or grouped to compare each homozygous or heterozygous genotype to other ones (only significant results are shown). Univariate analyses were repeated on subgroups of the population according to ER tumor status.

Multivariate analysis was performed on whole population combining significant SNP in a stepwise multivariate logistic regression selecting variables according to the Akaike Information Criterion (AIC). The model was validated internally on 1,000 random samples with replacement on the whole dataset. The percentage of times each variable was selected was extracted. Only those variables which were selected in >80% of models were retained. The final model was adjusted for ER status.

Results of statistical tests were considered significant at the 5% level. Analyses were performed using v11.2 Stata software (StataCorp. 2009. Stata Statistical Software: Release 11. College Station, TX, USA) and v2.15.2 R software (R Core Team 2012).

## Results

### Association between pCR and clinicopathological characteristics

25 patients (21.2%, CI 95%: 14.2–29.7%) reached complete pathologic response according to Sataloff criteria. Median age at diagnosis was 46 years for responder patients, 47 years for non-responder patients and was not significantly correlated to pCR.

The associations between pCR and clinicopathological features are summarized in Table 2. No statistically significant correlations were observed between pCR and tumor size, nodal status or KI67. Patients with higher tumor grade were significantly more likely to achieve a pCR than those with lower tumor grade (p = 0.009). We also observed different pCR rates according to Hormone Receptors (HR) status: patients whose tumors were positive for at least one HR had a pCR rate of 15.6% whereas patients who were HR negative had a pCR rate of 33.3% (p = 0.003). Contrary to PR negativity, ER negativity is a significant marker of pCR (p = 0.005). pCR rate is higher for ER− tumors (35.7%) than for ER+ tumors (13.5%; p = 0.005). Tumors with triple negative phenotype achieved significantly higher pathologic response rate than those with non-triple negative phenotype (40.6 vs 16.0%, p = 0.006).

**Table 2:** Association between clinicopathological features and pCR in FEC-Taxotere treated group (N = 118)

	Total	pCR (N = 25)		No pCR (N = 93)		P value
	N = 118	n	%^a^	n	%^a^	
*Tumor size*						
T0-T1	5	1	20.0	4	80.0	
T2	60	16	26.7	44	73.3	
T3	24	3	12.5	21	87.5	
T4	27	4	14.8	23	85.2	NS^†^
NA	2	1		1		
*Histopronostic grade*						
SBRI–SBRII	61	9	14.8	52	85.2	
SBRIII	43	16	37.2	27	62.8	0.009*
NA	14	0		14		
*Nodal status*						
Positive	63	12	19.1	51	80.9	
Negative	53	12	22.6	41	77.4	NS^†^
NA	2	1		1		
*KI67*						
Positive	42	12	28.6	30	71.4	
Negative	6	0	0.0	6	100.0	NS^†^
NA	70	13		57		
*Hormone receptor status*						
RH						
Positive^b^	77	12	15.6	65	84.4	
Negative^c^	39	13	33.3	26	66.7	0.003*
NA	2	0		2		
RE						
Positive	74	10	13.5	64	86.5	
Negative	42	15	35.7	27	64.3	0.005*
NA	2	0		2		
RP						
Positive	57	10	17.5	47	82.5	
Negative	58	15	25.9	43	74.1	NS*
NA	3	0		3		
*Triple negative*						
Yes	32	13	40.6	19	59.4	
No	75	12	16.0	63	84.0	0.006*
NA	11	0		11		

*NA* not applicable, *pCR* pathological complete response.

* Khi-2 test.

^†^ Fisher exact test.

^‡^ Wilcoxon Mann–Whitney test.

^a^Percentages of evaluable patients.

^b^Estrogen receptor or progesterone receptor positive.

^c^Estrogen receptor and progesterone receptor negative.

### Association between pCR and the 37 selected SNPs

Among the 37 SNPs evaluated, genotyping data were available for 114 of the 118 patients enrolled in the study. The distribution of the genotypes and allelotypes indicated that all frequencies were in Hardy–Weinberg equilibrium and were consistent with previously reported Caucasian populations (data not shown).

Univariate analysis revealed that 4 SNPs are significantly associated with pCR (Table 3).

**Table 3:** pCR is related to 4 SNPs (N = 114)

Gene	SNP		Genotype	N	pCR (N = 25)		OR	95% CI	P value
	rs #	wt			n	%^a^			
*BRCA1*	rs799917	C	CC	44	5	11.4	1		
	Pro871leu		CT-TT	66	19	28.8	3.15	(1.08–9.2)	0.025
			NA	4	1				
*SLCO1B3*	rs11045585	A	AA	79	13	16.5	1		
	IVS12-5676		AG-GG	34	12	35.3	2.77	(1.10–6.7)	0.031
			NA	1	0				
*CYP1B1*	rs1056836	C	CG	45	5	11.1	1		0.020
	Leu432Val		CC-GG	69	20	29.0	3.26	(1.12–9.5)	
*ERCC1*	rs11615	T	TT	40	4	10.0	1		
	Asn118Asn		CT-CC	73	21	28.8	3.63	(1.15–11.5)	0.016
			NA	1	0				

*NA* not applicable, *pCR* pathological complete response.

^a^Percentages of evaluable patients.

Patients carrying at least one variant allele for *BRCA1* (T), ERCC1 (C) and *SLCO1B3* (G) have about a threefold higher likelihood to achieve a pCR to NCT than other patients. Patients homozygous for *CYP1B1* Leu432Val showed increased pCR rate compared to heterozygous patients (respectively pCR = 29% and pCR = 11.1%, p = 0.020).

Subsequently, assessment of associations between combinations of these SNPs and pCR showed that a model containing only *ERCC1* and *CYP1B1* polymorphisms performed as well as more complex models. Stepwise logistic regression combining the four significant SNPs identified the model containing *CYP1B1* and *ERCC1* as the best: these SNPs were validated respectively in 81.0 and 80.8% of the models whereas *BRCA1* and *SLCO1B3* were selected in 69.2 and 72.3% of the models. The *ERCC1* CT genotype associated with *CYP1B1* CC genotype had the highest OR of 8.5 (p = 0.013; Table 4). pCR rate reached 50% for patients harboring these genotypes whereas the pCR rate is 21.2% in the whole population.

**Table 4:** pCR is related to combination of *CYP1B1* and *ERCC1* polymorphisms

CYP1B1 (Leu432Val)	ERCC1 (Asn118Asn)	N	pCR		OR	95% CI	P value
			n	%^a^			
CC	TT	19	2	10.5	1	Reference	
*CC*	*CT*	*22*	*11*	*50.0*	*8.5*	*(1.6–46)*	*0.013*
CC	CC	6	1	16.7	1.7	(0.13–23)	0.69
CG	TT	15	1	6.7	0.61	(0.05–7.4)	0.70
CG	CT	20	2	10.0	0.94	(0.12–7.5)	0.96
CG	CC	10	2	20.0	2.1	(0.25–18)	0.49
GG	TT	6	1	16.7	1.7	(0.13–23)	0.69
GG	CT	8	3	37.5	5.1	(0.66–39)	0.12
GG	CC	7	2	28.6	3.4	(0.38–31)	0.28

Significant results are shown in italic.

*pCR* pathological complete response.

^a^ Percentages of evaluable patients.

### Association between pCR and SNPs considering ER status

In breast cancer, tumor subtypes are associated with different responses to neoadjuvant therapies. For example, highly proliferative ER− breast tumors are more sensitive to NCT than ER+ breast tumors: pCR rates are respectively 28–32 and 2–10% (von Minckwitz 2013; Colleoni et al. 2004; Kaufmann et al. 2012).

As pCR can be achieved only in a minority of patients with ER+ breast tumors, it is of particular interest to identify patients who can avoid neoadjuvant chemotherapy or who are candidates for a very high probability of pCR after chemotherapy. Therefore, among the four SNPs previously described as significantly associated to pCR, we searched for potential predictive marker for ER+ breast tumors. pCR is significantly increased for patients carrying variant alleles of *BRCA1*, *SLCO1B3* and *CYP1B1* (Table 5, genotyping data obtained for 71 patients).

**Table 5:** *BRCA1*, *SLCO1B3* and *CYP1B1* are significantly associated with pCR in ER + tumors (N = 71)

Gene	SNP		Genotype	N	pCR (N = 10)		OR	95% CI	P value
	rs #	wt			n	%^a^			
*BRCA1*	rs799917	C	CC	26	0	0.0			
	Pro871Leu		CT-TT	42	9	21.4			*0.010*
			NA	3	1				
*SLCO1B3*	rs11045585	A	AA	49	4	8.2	1		
	IVS12-5676		AG-GG	21	6	28.6	4.50	(1.12–18.1)	*0.032*
			NA	1	0				
*CYP1B1*	rs1056836	C	CG	26	0	0.0			
	Leu432Val		CC-GG	45	10	22.2			*0.011*
*ERCC1*	rs11615	T	TT	22	1	1.5			
	Asn118Asn		CT-CC	48	9	18.8			0.08
			NA	1	0				

Significant results are shown in italic.

*NA* not applicable, *pCR* pathological complete response.

^a^Percentages of evaluable patients.

Considering patients with ER− tumors, only *ERCC1* (Asn118Asn) is significantly associated with response to NCT (Table 6, genotyping data obtained for 41 patients). Patients carrying at least one variant allele for *ERCC1* (C) have a fourfold higher likelihood to achieve a pCR after NCT than patients with other genotypes.

**Table 6:** *ERCC1* is significantly associated with pCR in ER- tumors (N = 41)

Gene	SNP		Genotype	N	pCR (N = 15)		OR	95% CI	P value
	rs #	wt			n	%^a^			
*BRCA1*	rs799917	C	CC	17	5	29.4			
	Pro871leu		CT-TT	23	10	43.5			0.36
			NA	1	0				
*SLCO1B3*	rs11045585	A	AA	28	9	32.1			
	IVS12-5676		AG-GG	13	6	46.2			0.39
*CYP1B1*	rs1056836	C	CG	18	5	27.8			
	Leu432Val		CC-GG	23	10	43.5			0.29
*ERCC1*	rs11615	T	TT	17	3	17.6	1		
	Asn118Asn		CT-CC	24	12	50.0	4.7	(1.06–20)	*0.030*

Significant results are shown in italic.

*NA* not applicable, *pCR* pathological complete response.

^a^Percentages of evaluable patients.

Moreover, combined *ERCC1*-CT and *CYP1B1*-CC genotypes remain significant when adjusted on ER status (p = 0.005; Table 7). In consequence, pCR rate reached 41.2% for patients with ER+ tumors harboring these genotypes whereas the pCR rate is only 13.5% for unselected patients with ER+ tumors. pCR rate is twice higher for patients with ER- tumors harboring combined *ERCC1*-CT and *CYP1B1*-CC genotypes than for unselected patients (80 vs 37.5%).

**Table 7:** pCR is related to combination of *CYP1B1* and *ERCC1* polymorphisms when adjusted on ER status

CYP1B1 (Leu432Val)	ERCC1 (Asn118Asn)	N	pCR		OR	95% CI	P value
			n	%^a^			
CC	TT	19	2	10.5	1	Reference	
CC	CT	22	11	50.0	16.1	(2.3–112)	*0.005*
CC	CC	6	1	16.7	2.8	(0.16–47)	0.48
CG	TT	15	1	6.7	0.41	(0.03–5.6)	0.51
CG	CT	20	2	10.0	0.77	(0.09–6.8)	0.81
CG	CC	10	2	20.0	2.4	(0.24–25)	0.45
GG	TT	6	1	16.7	0.89	(0.06–13.5)	0.93
GG	CT	8	3	37.5	4.4	(0.46–41)	0.20
GG	CC	7	2	28.6	3.1	(0.28–34)	0.35
ER−		42	15		1	Reference	
ER+		71	10		0.12	(0.03–0.43)	*0.001*

Significant results are shown in italic.

*pCR* pathological complete response.

^a^Percentages of evaluable patients.

## Discussion

In order to identify breast cancer patients who will benefit from anthracyclines and/or taxanes based NCT, we analyzed the relationship between 37 SNPs and the response to these chemotherapeutic agents.

First overall, our data confirmed that response to NCT is related to clinicopathological features such as histoprognostic tumor grade and hormone receptor status. According to previous published data (von Minckwitz 2013), we observed that pCR rate is increased in SBRIII and HR negative tumors and that ER- tumors respond better to NCT than ER+ tumors. Our data also indicated that patients with triple negative tumors achieve significantly higher pathologic complete response rate than those with non-triple negative tumors. Unexpectedly, pCR was not significantly associated to KI67 expression in our study. Nevertheless, this observation may be explained in part by the fact that data were missing for more than half of patients.

Secondly, considering the predictive value of the 37 studied SNPs, we demonstrated using univariate analyses that *BRCA1* (Pro871Leu) and *ERCC1* (Asn118Asn), two polymorphisms located in DNA repair genes, and also *CYP1B1* (Leu432Val) and *SLCO1B3* (IVS12-5676), two polymorphisms in genes involved in drug metabolism, are linked to response to taxanes and anthracyclines. Multivariate analysis revealed that *ERCC1* (Asn118Asn) combined to *CYP1B1* (Leu432Val) might be of particular interest as predictive markers for NCT in breast cancer. Indeed, we observed that patients exhibiting *ERCC1*-CT genotype combined to *CYP1B1*-CC genotype presented a doubling of pCR rate compared with unselected patients (50 vs 21.2%).

Our results are in agreement with previous published data. The *BRCA1* variant allele (T) is associated with better overall survival and longer progression-free survival compared to the reference allele in patients with advanced gastric cancer treated with taxanes and cisplatin (Shim et al. 2010).

Although considered as neutral, the functional significance of Pro871Leu (C > T) polymorphism of *BRCA1* is still unknown. Pro871Leu might affect expression, activity or interaction of *BRCA1* with its partners. As a consequence, Pro871Leu might impair DNA repairing function of *BRCA1* and might contribute to potentiate efficacy of DNA damaging agents.

*ERCC1* CC genotype was reported to confer longer survival and time to progression in patients with NSCLC treated with cisplatin plus docetaxel (Isla et al. 2004). Any other study described a link between *ERCC1* polymorphism and response to taxanes/anthracyclines, probably because *ERCC1* polymorphism has extensively been studied in response to platinium compounds, *ERCC1* C genotype being related to both a better (Isla et al. 2004; Chang et al. 2009; Kalikaki et al. 2009; Warnecke-Eberz et al. 2009; Metzger et al. 2012) and a worse response (Viguier et al. 2005; Kamikozuru et al. 2008; Ren et al. 2012). The consequence of the silent polymorphism Asn118Asn (T > C) on *ERCC1* transcription and expression is not elucidated and remains still conflicting. Gao & co had demonstrated no difference in *ERCC1* transcription nor expression between cells stably expressing *ERCC1*-T allele or *ERCC1*-C allele (Gao et al. 2011). However, data obtained on lymphocytes of prostate cancer patients revealed that carriers of CC genotype showed lower *ERCC1* mRNA levels (Woelfelschneider et al. 2008).

Thus, by decreasing *ERCC1* mRNA levels, C genotype might decrease *ERCC1* mediated DNA repair and might contribute to potentiate efficacy of DNA damaging drugs. This hypothesis is in agreement with clinical data which present that low levels of *ERCC1* are favorable for sensitivity to platinium compounds (Li et al. 2000; Shirota et al. 2001; Lord et al. 2002; Wang et al. 2008).

*CYP1B1* Val genotype is associated with lower response rate, shorter progression-free-survival and decrease overall survival in breast and prostate cancer patients treated with taxanes based chemotherapy (Marsh et al. 2007; Sissung et al. 2008; Pastina et al. 2010). However, in our study, only Leu/Val genotype provided the best data fit for the SNP Leu432Val. The hypothesis of an overdominant model, previously described in bladder cancer (Salinas-Sanchez et al. 2012), might be explained by a higher enzyme activity in heterozygous than in homozygous. *CYP1B1* binds and sequestrates docetaxel reducing its available concentration and influencing its activity (McFadyen et al. 2001). *CYP1B1* also produces oestrogen metabolites that limit docetaxel efficacy by inhibition of tubulin polymerization (Sissung et al. 2008). Thus, by increasing *CYP1B1* mRNA expression and catalytic activity (Shimada et al. 1999; Hanna et al. 2000; Li et al. 2000; Landi et al. 2005), Leu432Val substitution appears to modulate response to taxanes.

*SLCO1B3* is an influx transporter involved in the uptake of a broad range of drug substrates including docetaxel (Konig et al. 2000; Smith et al. 2005). The functional impact of rs11045585 polymorphism is yet unknown. However, asian nasopharyngeal cancer patients homozygous for the variant allele had higher area under curve and less plasma clearance of docetaxel compared to patients carrying at least one reference allele (Chew et al. 2011). It is to be noted that rs11045585 polymorphism is proposed to exert its functional effect by being in haplotypic combination with 3 other polymorphism in the *SLCO1B3* gene (Chew et al. 2012).

Finally, as pCR is rarely achieved by NCT in ER+ breast tumors, we searched if *BRCA1* (Pro871Leu), *ERCC1* (Asn118Asn), *CYP1B1* (Leu432Val) and *SLCO1B3* (IVS12-5676) could stand for predictive markers of NCT for patients with this subtype of tumor. Our results indicated that *BRCA1* (Pro871Leu), *CYP1B1* (Leu432Val) and *SLCO1B3* are significantly associated with response to NCT in ER+ tumors. *ERCC1/CYP1B1* combination remains significant for ER+ tumors and might be useful to help indication of NCT in this particular type of tumors as patients exhibiting *ERCC1*-CT genotype combined to *CYP1B1*-CC genotype presented a threefold higher pCR rate compared with unselected patients: 41.2 vs 13.5%.

Our data also indicated that *ERCC1* Asn118Asn might be a potential predictive marker for NCT in ER- tumors, as pCR is increased to 50% for patients carrying at least one variant allele (C).

In conclusion, *BRCA1* (Pro871Leu), *ERCC1* (Asn118Asn), *CYP1B1* (Leu432Val), and *SLCO1B3* (rs11045585) are associated with response to NCT in our cohort of breast cancer patients. To our knowledge, this study is the first to report *ERCC1*, *BRCA1* and *SLCO1B3* as markers of taxanes and/or anthracyclines response in breast cancer NCT.

Moreover *ERCC1*/*CYPB1* combination appeared to be a potential predictive marker to guide NCT indication in breast cancer and particularly for ER+ breast tumors.

## Additional file

Additional file 1.
**Table S1:** List of polymorphisms selected for the study.
