# Supplementary material for: ERCC1 and CYP1B1 polymorphisms as predictors of response to neoadjuvant chemotherapy in estrogen positive breast tumors
Source: Springerplus. 2015 Jul 7;4:327. doi: 10.1186/s40064-015-1053-0 (PMC4493257; doi:10.1186/s40064-015-1053-0)
Supplement: Additional file 1. — Table S1: List of polymorphisms selected for the study. [file 40064_2015_1053_MOESM1_ESM.doc]

Supplementary Table 1. List of polymorphisms selected for the study

| **Gene** | **ref SNP** | **References**  **Supplemental Table 1.** |
| --- | --- | --- |
| ***ABCC4*** | rs9561778 | Low SK, Kiyotani K, Mushiroda T et al (2009). Journal of Human Genetics 54 : 564-571 |
| ***BRCA1*** | rs799917 | Shim HJ, Yun JY, Hwang JE et al (2010). Cancer Science 101 : 1247-1254 |
| ***CES2*** | rs11075646 | Ribelles N, Lopez-Siles J, Sanchez A et al (2008). Current Drug Metabolism 9 : 336-343 |
| ***CD24*** | rs52812045 | Marmé F, Werft W, Walter A et al (2012). Breast Cancer Research 132: 819-831 |
| ***CYP1B1*** | rs1056836 | Marsh S, Somlo G, Li X et al (2007). The Pharmacogenomics Journal 7: 362-365  Rizzo R, Spaggiari F, Indello M et al (2010). Breast Cancer Research and Treatment 124: 593-598 |
| ***CYP2B6*** | rs8192709 | Bray J, Sludden J, Griffin MJ et al (2010). British Journal of Cancer 102: 1003-1009 |
|  | rs12721655 |  |
|  | rs3745274 |  |
|  | rs2279343 |  |
|  | rs7254579 | Nakajima M, Komagata S, Fujiki Y et al (2007). Pharmacogenetics and Genomics 17: 431-445 |
|  | rs4802101 |  |
|  | rs4803419 |  |
|  | rs2279345 |  |
| ***CYP3A4*** | rs2740574 | Gor PP, Su HI, Gray RJ et al (2010). Breast Cancer Research 12: R26 |
| ***DNASE2B*** | rs3738573 | Ha HJ, Yoon SN, Jeon YJ et al (2011). Anticancer Research 31: 4329-4338 |
| ***DPD*** | rs2297595 | Gross E, Busse B, Riemenschneider M et al (2008). PLoS One 3 : e4003 |
| ***ERCC1*** | rs11615 | Isla D, Sarries C, Rosell R et al (2004). Annals of Oncology 15: 1194-1203 |
| ***FGFR4*** | rs351855 | Marmé F, Werft W, Benner A et al (2010). Annals of Oncology 21: 1636-1642 |
| ***GSTP1*** | rs1695 | Oliveira AL, Rodrigues FF, Santos RE et al (2010). Genetics and Molecular Research 9: 1045-1053  Yao S, Barlow WE, Albain KS et al (2010). Clinical Cancer Research 16: 6169-6176  Romero A, Martin M, Oliva B et al (2012). Annals of Oncology 23: 1750-1756 |
| ***HTRIE*** | rs3828741 | Ha HJ, Yoon SN, Jeon YJ et al (2011). Anticancer Research 31: 4329-4338 |
| ***IL6*** | rs1800795 | DeMichele A, Gray R, Horn M et al (2009). Cancer Res 69: 4184-4191 |
|  | rs1800797 |  |
| ***MDR1*** | rs2032582 | Bray J, Sludden J, Griffin MJ et al (2010). British Journal of Cancer 102: 1003-1009  Chang H, Rha SY, Jeung HC et al (2009). Annals of Oncology 20: 272-277 |
|  | rs1045642 | Kafka A, Sauer G, Jaeger C et al (2003). International Journal of Oncology 22: 1117-1121  Cizmarikova M, Wagnerova M, Schonova L et al (2010). The Pharmacogenomics Journal 10: 62-69  Fajac A, Gligorov J, Rezai K et al (2010). British Journal of Cancer 103: 560-566 |
|  | rs2229109 |  |
| ***MTHFR*** | rs1801131 | Zarate R, Gonzalez-Santigo S, de la Haba J et al (2007). Current Drug Metabolism 8 : 481-486 |
|  | rs1801133 | Henriquez-Hernandez LA, Murias-Rosales A, Gonzales-Hernandes A et al (2010). Cancer Epidemiology 34 : 634-638 |
| ***NOS3*** | rs1799983 | Choi JY, Barlow WE, Albain KS et al (2009). Clinical Cancer Research 15: 5258-5266 |
|  | rs2070744 |  |
| ***NQO1*** | rs1800566 | Fagerholm R, Hofstetter B, Tommiska J et al (2008). Nature Genetics 40: 844-853  Jamieson Cresti N, Bray J et al (2011). Pharmacogenetics and Genomics 21: 808-819 |
| ***NQO2*** | rs1143684 | Jamieson D, Cresti N, Bray J et al (2011). Pharmacogenetics and Genomics 21 : 808-819 |
| ***p53*** | rs1042522 | Xu Y, Yao L, Ouyang T et al (2005). Clinical Cancer Research 11 : 7328-7333  Henriquez-Hernandez LA, Murias-Rosales A, Gonzales-Hernandes A et al (2010). Cancer Epidemiology 34 : 634-638 |
| ***PARP1*** | rs1136471 | Shiraishi K, Kohno T, Tanai C et al (2010). Journal of Clinical Oncology 28: 4945-4952 |
| ***SHMT1*** | rs1979277 | Budai B, Komlósi V, Adleff V et al (2012). Pharmacogenetics and Genomics 22: 69-72 |
| ***SLCO1B3*** | rs11045585 | Chew SC, Singh O, Chen X et al (2011). Cancer Chemotherapy and Pharmacology 67: 1471-1478 |
| ***SOD2*** | rs4880 | [Glynn SA](http://www.ncbi.nlm.nih.gov/pubmed?term=Glynn SA%5BAuthor%5D&cauthor=true&cauthor_uid=19509150), [Boersma BJ](http://www.ncbi.nlm.nih.gov/pubmed?term=Boersma BJ%5BAuthor%5D&cauthor=true&cauthor_uid=19509150), [Howe TM](http://www.ncbi.nlm.nih.gov/pubmed?term=Howe TM%5BAuthor%5D&cauthor=true&cauthor_uid=19509150) et al (2009). Clinical Cancer Research 15: 4165- 4173  Yao S, Barlow WE, Albain KS et al (2010). Breast Cancer Research and Treatment 124: 433-439 |
| ***XRCC1*** | rs25487 | [Jaremko M](http://www.ncbi.nlm.nih.gov/pubmed?term=Jaremko M%5BAuthor%5D&cauthor=true&cauthor_uid=17558308), [Justenhoven C](http://www.ncbi.nlm.nih.gov/pubmed?term=Justenhoven C%5BAuthor%5D&cauthor=true&cauthor_uid=17558308), [Schroth W](http://www.ncbi.nlm.nih.gov/pubmed?term=Schroth W%5BAuthor%5D&cauthor=true&cauthor_uid=17558308) et al (2007). Pharmacogenetics and Genomics 17: 529-533 |
